# Untargeted Metabolomics Analysis for Studying Differences in High-Quality Colombian Cocoa Beans

**DOI:** 10.3390/molecules28114467

**Published:** 2023-05-31

**Authors:** Paula Bacca-Villota, Luis Acuña-García, Leidy Sierra-Guevara, Herminsul Cano, William Hidalgo

**Affiliations:** Escuela de Química, Universidad Industrial de Santander, Bucaramanga 680002, Colombia; paulabv89@hotmail.com (P.B.-V.); luis.acuna@correo.uis.edu.co (L.A.-G.); leidy.sierra3@correo.uis.edu.co (L.S.-G.); hjcanoc@uis.edu.co (H.C.)

**Keywords:** cocoa beans, metabolomics, quality and flavor, principal component analysis (PCA), UHPLC/HRMS

## Abstract

Colombia is a producer of fine cocoa, according to the International Cocoa Organization; however, most of its exports are in the ordinary cocoa category. To remedy this situation, several national organizations are working to create technological platforms for small producers to certify the quality of their beans. The objective of this study was to identify differential chemical markers in 36 cocoa bean samples from five Colombian departments and associate them with cocoa quality properties. For this purpose, a non-targeted metabolomics approach was performed using UHPLC-HRMS, along with sensory and physicochemical analyses. The 36 samples did not differ in sensory quality, polyphenol content, and theobromine/caffeine ratio. However, the multivariate statistical analysis allowed us to differentiate the samples into four clusters. In addition, a similar grouping of the samples was also observed in the physical analyses. The metabolites responsible for such clustering were investigated with univariate statistical analysis and presumptively identified by comparison of experimental mass spectra with those reported in databases. Alkaloids, flavonoids, terpenoids, peptides, quinolines, and sulfur compounds were identified as discriminants between sample groups. Here, it was presented the metabolic profiles as an important chemical feature for further studies in quality control and more specific characterization of fine cocoa.

## 1. Introduction

Cocoa (*Theobroma bovalifolium*) is a commodity product that ranks third in the global market, after sugar and coffee [1]. In Colombia, cocoa bean production is one of the most important agricultural activities, since in 2021, more than 65,000 tons of grains were produced, and export trading reached a value of more than USD 29 million [2]. In Colombia, most cocoa bean production comes from the Criollo variety, which has attractive sensory properties [3]. According to the Cocoa International Organization (ICCO), Colombia produces fine or special cocoa, which highlights its fine grain, soft texture, and excellent aroma. However, most of the production is destined for local consumption, and a reduced amount that reaches international markets does it in the category of regular cocoa [4]. One of the main subjects to change to improve Colombia’s participation in the global trading of special cocoa is the implementation of emerging technologies that allow small cocoa producers to certify the high quality of their products. The quality of the cocoa is an important feature of the value chain since it will determine the demand and added value to the final product. Currently, the quality of cocoa beans is determined by a set of physical, chemical, and sensory properties such as cadmium content, fermentation degree, polyphenols content, theobromine and caffeine amounts, and flavor notes [5].

Previous studies about the chemical composition of cocoa beans have identified both volatile and non-volatile compounds that keep a relationship with sensory properties; secondary metabolites such as polyphenols and methylxanthines, along with carbohydrates and proteins, are assigned to be responsible for the taste and aroma of cocoa beans [6]. Polyphenols are another group of compounds with high occurrence in cocoa beans; recently, these organic compounds have aroused interest in the scientific community due to their antioxidant properties, which also confer astringent and bitter sensations and contribute significantly to the green and fruity flavors of cocoa liquors [7]. Some important polyphenols present in cocoa are epicatechin, catechin, and proanthocyanidins B1 and B2 [8]. Xanthine and methylxanthines are other important classes of non-volatile compounds. The three main xanthine-alkaloids in cocoa are theophylline, theobromine, and caffeine [9]. Some studies point out that the theobromine/caffeine ratio in cocoa beans can be a marker of “fineness”, and other properties such as the fermentation state of the bean and flavor descriptors such as bitterness have been found to keep a relationship with this coefficient [10,11].

In recent years, metabolomics has emerged as a tool to study the chemical fingerprinting of some natural products and their relationship with desirable or exploratory parameters. For cocoa, targeted metabolomics approaches have been applied to identify and quantify compounds of interest, such as polyphenols, methylxanthines, catechins, and flavonoids [9]. Other studies have also sought to relate these compounds to the geographical origin of cocoa samples [12], the evaluation of the fermentation state of the grain [13], and the study of the dynamics of its volatile and non-volatile compounds [14]. However, few studies have been concerned with identifying possible chemical markers present in cocoa samples that may correlate with desirable sensory properties and cocoa bean quality [15,16]. Considering the above, non-targeted metabolomics appears to be a useful tool, as it facilitates the analytical task of identifying multiple metabolites in chemically diverse matrices, such as cocoa beans, in an easier and faster method.

Therefore, this study aimed to identify differential chemical markers in samples of premium cocoa beans and investigate their relationship with sensory properties associated with cocoa quality. It is expected that chemical markers will contribute to developing a better technological platform for certification of the quality of cocoa beans from small producers in Colombia, which emerges as one of the most attractive alternatives to substituting illicit crops [17].

## 2. Results and Discussion

### 2.1. Physicochemical Characterization of Cocoa Samples

Cocoa beans corresponded to fermented and dried beans from different farms located in Maceo (Antioquia), Manaure (Cesar), Santa Marta (Magdalena), San Vicente de Chucurí (Santander), and Norte de Santander, Colombia, which were selected according to the identification of the sample (or lot), processing plant, number of cocoa trees, and educational level of the producer. A total of 36 cocoa samples were collected, and they were classified as fine or flavor cocoa (*n* = 28) and ordinary cocoa (*n* = 8; REG, CCN 1, L8T7, CCN 12, MAN 5, SV 1, MON, and ICS); furthermore, 15 of the 27 samples of fine or flavor cocoa are currently exported to North American and European markets (Supplementary Materials, Table S1).

The world cocoa market classifies cocoa beans into two broad categories: “fine” or “flavor” cocoa beans and “bulk” or “ordinary” cocoa beans [18]; fine cocoa beans come from Criollo or Trinitario cacao varieties, while ordinary cocoa beans come from Forastero trees [19]. This classification has several exceptions, so it is currently considered that there is no single universal criterion, and therefore, a combination of the following criteria is used to evaluate the quality of cocoa: the genetic origin of the planting material, the morphological characteristics of the plant, the flavor characteristics of the cocoa beans produced, the chemical characteristics of the cocoa beans, the degree of fermentation, drying, acidity, off-flavors, percentage of internal mold, insect infestation, and percentage of impurities [20]. According to the classification of ICCO, Colombia has 95% of its cocoa grown as fine or flavor [19]. However, a large percentage is still exported under the conventional category [19].

#### 2.1.1. Physical Analysis

The physical characterization of the cocoa samples is summarized in Table 1. The grain index, total defective grains (moldy, insect-damaged, sprouted, and slaty grains), fermentation degree (unfermented, partially fermented, fermented, and over-fermented grains), cracking degree, and the global physical rating (0.0 is the lowest value and 10.0 is the highest value) were evaluated, and the results showed that all samples had a global physical rating greater than 7.9.

The grain index is the average bean weight in grams (g) taken from a sample of 100 dry cocoa beans (g/grain). This weight is expected to be greater than 100 g to ensure a suitable pod index (average number of pods needed to obtain 1 kg of dry cocoa). According to Federación Nacional de Cacaoteros (FEDECACAO), a bean index greater than 1.7 g/grain is high, between 1.7 and 1.4 g/grain is medium, and less than 1.4 g/grain is low [21]. In this study, 50% of the samples had a medium grain index, and the remaining percentage had a low grain index. The cracking degree is caused by proteolysis during fermentation and is related to the internal structure of a grain. Generally, the relationship between cracking and fermentation degree is directly proportional since insufficient fermented or roasted kernels adhere to the hull of the almond, making it difficult to separate them [20]. According to the Norma Técnica Colombiana NTC-1252, fine or flavored cocoa must have a percentage of well-fermented beans greater than (or equal to) 70% and ordinary cocoa higher than (or equal to) 65% [22]. In this study, all samples had a cracking degree higher than 70%, 2 samples (L8T7 and SV 1) had a fermentation degree less than 65%, 3 samples (CCN 1, CCN 4, and MEZ 1) were between 65% and 69%, and 31 samples had a fermentation degree greater than (or equal to) 70% (Table 1).

Defective grains are defined as grains with internal mold, insect-damaged grains, sprouted grains (seed germ has perforated the shell), or slaty grains (at least half of the surface of the cotyledons exposed by the shear test are slaty in color). The Food and Drug Administration (FDA) Standard stipulates a maximum of 4% moldy kernels and a maximum of 4% insect-infected or damaged kernels, but the total of these two must not exceed 6%. On the other hand, the percentage of sprouted and slaty grains must be lower than 10% [20]. The total number of defective grains reported in Table 1 is the sum of all defects (moldy, insect-infected, damaged, sprouted, and slaty). According to the results, the SDR sample exceeded the allowable range for moldy grains (12%) and sprouted (10.5%), and URA 3 also exceeded the allowable range for insect-damaged grains (21%) and slaty (11%). The remaining 34 samples fall into the allowable range for slaty, moldy, and infected kernels. However, from these 34 samples, 15 exceeded the permitted range for sprouted grains.

#### 2.1.2. Chemical Analysis

Cadmium concentration, total phenolic content, and theobromine/caffeine ratio constitute other parameters of relevance for determining the cocoa bean quality. In this sense, Table 2 shows the results of the chemical analyses for the cocoa samples assessed.

According to results, the total phenolic content varied between 88.1 and 168.2 mg G.A/g sample; additionally, 30 samples presented a value above 100 mg G.A/g sample, which is considered as high content for non-roasted grains [7]. An outcome such as the above is desirable, since polyphenols contribute to the astringent taste of cocoa, they are suitable antioxidants (inhibit lipid peroxidation by reducing free radicals and chelating metals) and they are responsible for the positive health benefits associated with cocoa consumption [7]. The value of the theobromine/caffeine (T/C) ratio for most of the samples was less than 4, and only sample 6 had a value of 4.8 (Table 2). T/C ratio in cocoa beans is another important indication of “fineness” [23]. Several authors have reported that this coefficient can be related to various properties of cocoa beans. Urbańska et al., 2019 and Carrillo et al., 2014, for example, found that the theobromine/caffeine ratio correlated with the place of origin of cocoa beans [24,25]; Brunetto et al., 2019 established this coefficient as related to cocoa genotype and variety [26], and Calvo et al., 2021 found this ratio to be useful as a biomarker of the fermentation state of the bean [10]. On the other hand, Ordoñez et al., 2020 established that the T/C ratio is related to bitterness, which is one of the descriptors of fineness in special cocoa beans [11]. Sotelo et al., 1991 reported a relation of T/C value for Forastero or “bulk” cocoa beans above 4, even higher than 10, while Criollo cocoa beans were lower than 4. Since all the samples used in our study corresponded to the Criollo variety (Supplementary Material, Table S1), the results obtained were aligned to those reported by other authors. However, there was no noticeable relationship between the theobromine/caffeine ratio and the geographical origin of the samples; this could be explained by the coexistence of different microclimates in geographically close areas and the climatic variability characteristic of different regions in Colombia.

Latin America currently accounts for about 15% of world cocoa production, and in Colombia, despite being a major producer of fine aroma cocoa (a term applied to a special quality production), regional cocoa farming is facing many challenges, including the presence of cadmium (Cd) in the soil, a heavy metal that accumulates in cocoa beans and it has been associated with harmful effects on human health [27,28]. European Commission Regulation No. 488/2014, which came in January 2019, fixed tolerable limits between 0.1 and 0.8 µg Cd g^−1^ to cocoa-derived products [29].

All samples of the study did fall into the permitted limits of Cd, except for SAR, SDR, URA 1, URA 2, and URA 3 cocoa samples, whose values exceeded the tolerable limits accepted; this can be attributed to several factors, including chemical, physical, and biological characteristics of the soil, contamination by use of agrochemicals or irrigation water. Another important factor is the assimilation capacity of cocoa trees, a characteristic that is influenced by conditions associated with the genotype of the plant. The problem represented by the presence of cadmium in cocoa has been the subject of numerous investigations carried out by various organizations and institutions in producing and consuming countries; in the case of Colombia, entities such as FEDECACAO and the Colombian Agricultural Research Corporation (AGROSAVIA) are concerned about this important issue in national cocoa production chain [28].

#### 2.1.3. Sensory Analysis

The sensory tests were performed by five tasters from the Cacaos Especiales de Antioquia y Cesar (CAESCA) panel, who were previously trained according to the method reported by Chetschik et al. [30]. Tasters evaluated different criteria of aroma, basic flavors, trigeminal sensation, and atypical flavors (Table 3) [30]. In this evaluation, the taster should consider that some criteria appear or disappear very quickly, while others may persist for a longer time. Then, the intensity of the attributes is scored for each descriptor, using a scale of categories from 0 “imperceptible” to 10 “intense” [30,31]. This denarius system can be divided into shorter ranges of scores that will have the following meanings: 0 = None present, 1 = Just a trace and may not be found if tasted again, 2 = Present in the sample, 3 to 5 = Clearly characterizing the sample, 6 to 8 = Dominant characterization of the sample, and 9 to 10 = Maximum (exceeds some other flavor notes in the sample) [32].

The aroma descriptors are used as an indicator of the dominant flavors that may be present in the liquor or cocoa products. The cocoa note relates to well-fermented, roasted, and defect-free cocoa beans. In the study, 13 samples had a cocoa note between 2.3 and 2.9 (present in the sample), and 23 samples had a cocoa note between 3.0 and 5.0 (clearly characterizing the sample). The fresh fruit descriptor is related to notes of citrus fruits (lemon, orange), tropical fruits (pineapple, passion fruit), and berries (raspberry, blackberry) [30]. Here, the number of samples with citrus fruit notes were 8 between 0.0 and 0.9 (none present), 18 between 1.0 and 1.9 (just a trace), 5 between 2.0 and 2.5 (present in the sample), and 5 between 3.0 and 4.4 (clearly characterizing the sample). The dried fruit attribute is associated with notes of apricots and raisins, among others [33]; within the group of samples, 8 were between 0 and 0.9 (none present), 27 between 1.0 and 1.9 (just a trace), and only 1 sample (SNM) was characterized by presenting this type of notes (2.3). The floral aroma, related to notes of orange blossom, lime blossom, and fresh rose, was only present in traces in samples CCN 3, URA 3, and POR 1 (values between 1.0 and 1.3). The wood aroma, related to notes of oak, teak, ash, or beech, was only present in traces in 5 samples CCN 1, CCN 4, CCN 8, CCN 13, and MAN 5 (values between 1.0 and 1.5). Spice refers to spicy notes such as cinnamon, clove, vanilla, bell pepper, or tobacco. These spices were found in traces in 14 samples (1.0–1.8) and were present in samples CCN 4 and MAN 3 (2.0–2.4). The nut descriptor is related to nutty notes such as almond, hazelnut, and peanut, among others. The results showed that the number of samples with nutty notes was 1 (CCN 12) with a value of 0.8 (none present), 14 between 1.3 and 1.9 (just a trace), 16 between 2.0 and 2.9 (present in the sample), and 5 between 3.0 and 3.3 (clearly characterizing the sample). The sweetness or caramel refers to notes of honey, panela, milk sweet, or cane juice [34]; 3 samples (L8T7, CCN 10, and CCN 12) were characterized by an absence of sweetness (0.7–0.8), 29 samples had traces of sweetness (1.0–1.9), and 4 samples (URA 1, CCN 4, CCN 13, and SV1) had sweetness (2.0–2.8). Finally, aroma related to cocoa toast intensity was present in 16 samples (2.2–2.9), with 19 samples (3.0–5.8) and only 1 sample (CCN 8) with a value of 6.8.

Flavor is a key criterion for determining the quality of cocoa products. This criterion includes the intensity of cocoa or chocolate flavor, along with other secondary aromatic notes, and the absence of undesirable flavors. The descriptors of basic tastes (acidity and bitterness), trigeminal sensation (astringency), and atypical flavors were used to qualify the flavor of the cocoa samples in this study (Table 4). Acid taste is related to acetic and lactic acids formed during fermentation. A high level of acidity is usually associated with a pH of 5.0 (or less) in dried grains, resulting in undesirable flavor and deficient cocoa product. Normally, when adequate drying is carried out, the acidity caused by acetic acid is reduced to an acceptable low value [20].

The acidity of the samples L8T7, CCN 3, CCN 10, and CCN 11 had the lowest value (1.5–1.9), while 14 samples were between 2.0 and 2.9 (level 2, present), clear characteristics were found for 13 samples (3.3–5.0) and dominant characteristic for SAR and MON (6.0–6.2). In relation to the degree of bitterness and astringency (contraction of the buccal mucosa caused by chemical stimuli such as polyphenols), it is considered that a certain value is part of the chocolate flavor complex, but its excessive presence is unpleasant and is generally associated with insufficient fermentation, slaty, and purple grains. This can be corrected by having a higher percentage of partially brown color rather than purple grains, with adequate harvesting, fermenting, and drying processes [35]. In this study, 1 sample (URA 1) had a bitterness of 1.8 (just a trace), 6 samples (POR 1, POR 2, SAR, MAK, URA 3, and SNM) were in the range of 2.3–2.5 (present in the sample), and 29 samples were between 3.0 and 5.3 (clearly characterizing the sample). Astringency values were between 1.3 and 1.9 for 21 samples (just a trace), between 2.0 and 2.5 for 12 of ours (present in the sample), and between 3.0 and 3.3 (clearly characterizing the sample) for samples MAN 1, CCN 4, and CCN 10. Finally, considering cocoa is a food-grade material, it is very important to perform proper handling to avoid the presence of undesirable flavors such as rot, mold, smoke, animal residues, copra, rubber, and fuels, among others [36]. This is due to the absorbent behavior of cocoa fat, which is very effective for all types of contamination [20]. Here, most of the samples did not present any outlier flavor, and only 5 samples had traces of outliers, L8T7 (1.9) reported an outlier flavor associated with plastic, CNN 12 (1.3) presented an unpleasant lingering bitter and overripe fruit flavor (rancid), REG, MON, and ICS (1.0) presented a small defect of rancid, stored, and vinegar. The overall score reflects the impression of quality in terms of cleanliness, intensity, complexity of aroma, and balance between flavors in the sample [37]. The results showed that 8 samples had a regular overall score between 4.9 and 6.8, and 28 samples had a high overall score greater than (or equal to) 7.0. L8T7 was the sample with the lowest score (4.9), probably related to the presence of atypical flavors in traces, and URA 1 was the sample with the highest score (8.8).

### 2.2. Untargeted Metabolic Profiling Analysis

**Figure 1:**
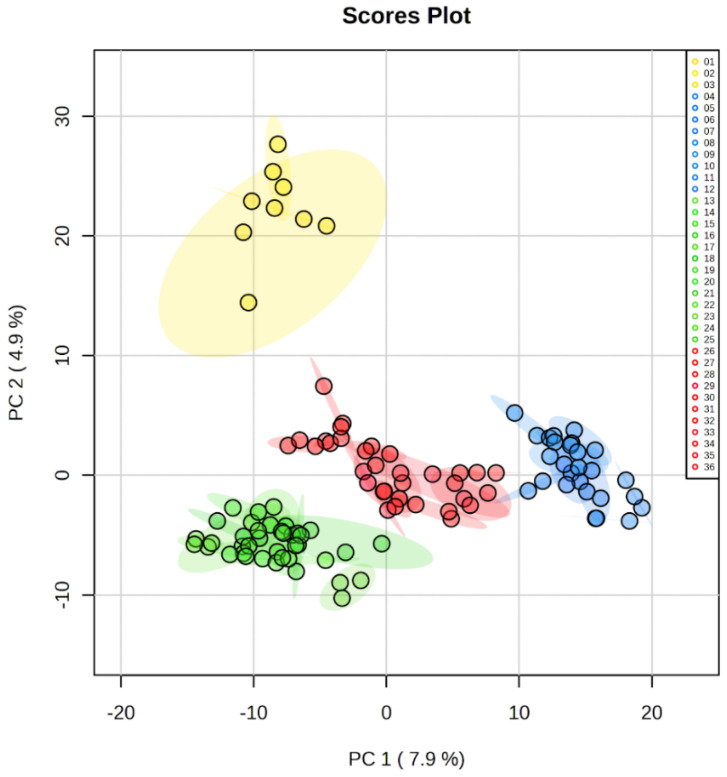
Principal component analysis (PCA) score plot of the metabolic profiles from cocoa beans samples after the identification of 4 groups. The samples with the closest values in the two first components were clustered into the same group. The four groups were differentiated by color: yellow (samples 01 to 03), blue (samples 04 to 12), green (samples 13 to 25), and red (samples 26 to 36). Each sample had a technical triplicate. To see sample identifier, refer to Table 4 and Supplementary Materials.

Preliminary analysis of the raw mass data performed on the XCMS platform allowed a total of 1307 (*m/z)* features across all samples; these data were filtered based on the coefficient of variation (% CV) of the peak chromatographic intensities, and 1204 features had a CV equal to or less than 25%. These data were arranged in a new matrix array, which constituted the metabolic profile of all samples studied. To study the chemical composition of the cocoa bean samples, a non-selective approach was applied, in which the metabolic profiles of the samples were analyzed by using uni- and multivariate statistical methods. Principal component analysis (PCA) was applied in the first instance to figure out chemical patterns in the samples (Figure 1).

Figure 1 shows the PCA score plot with a covariance of the samples through two coordinate axes or components; in this sense, the samples that were close in these components are likely to have similar chemical patterns and, therefore, present similar metabolic profiles. The first component (PC-1) accounted for 7.9% of the total variance, while PC2 explained 4.9%. These results showed a clear clustering of the samples around 4 main groups, which were classified on color-based coding as described in Figure 1.

**Figure 2:**
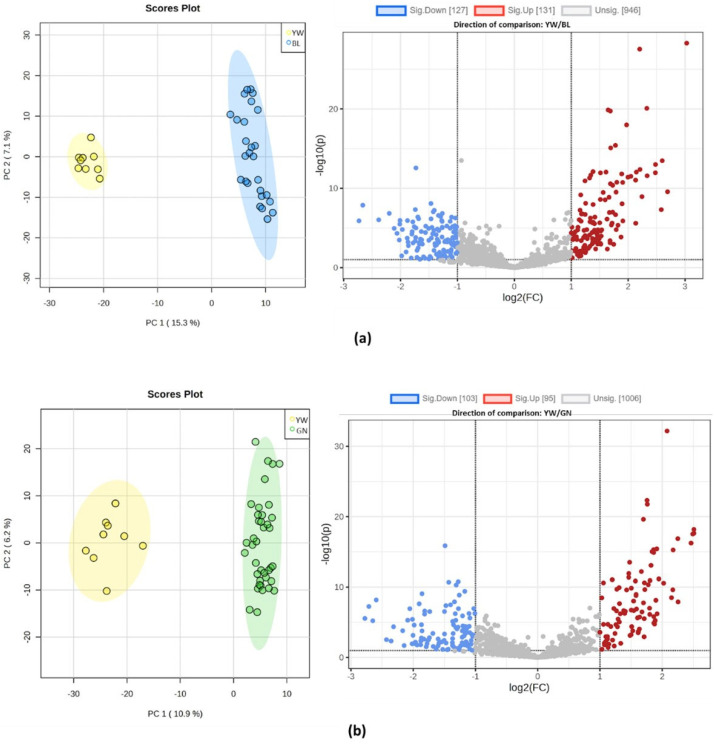

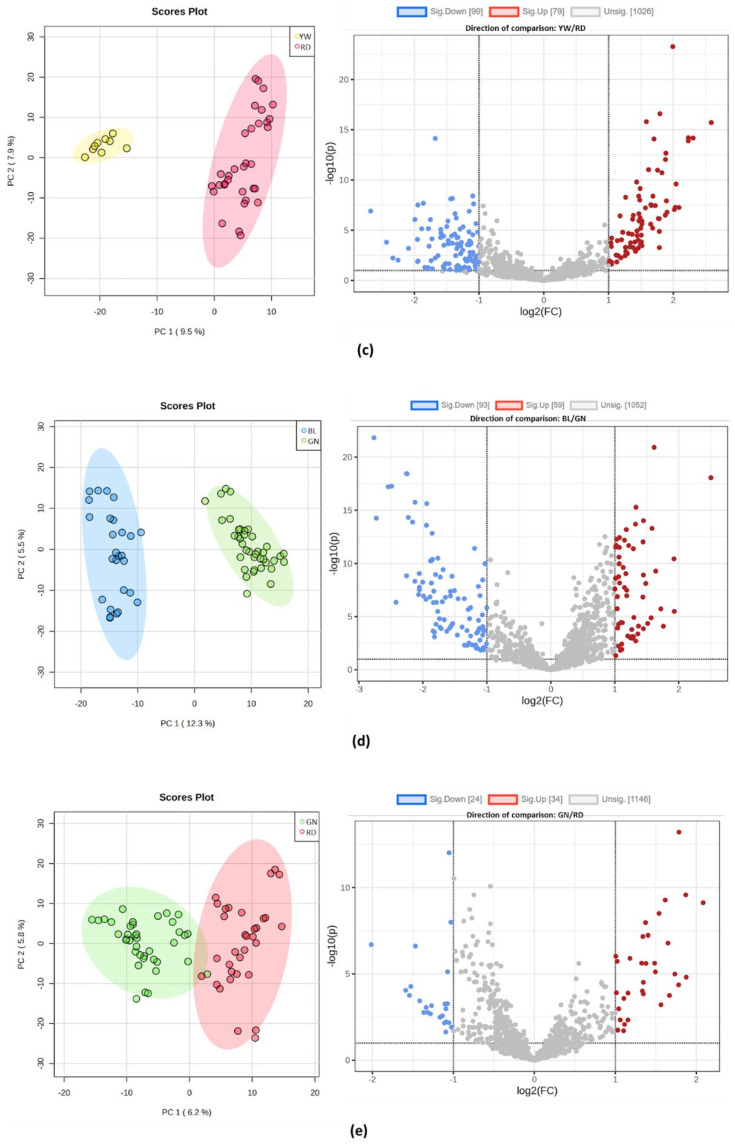

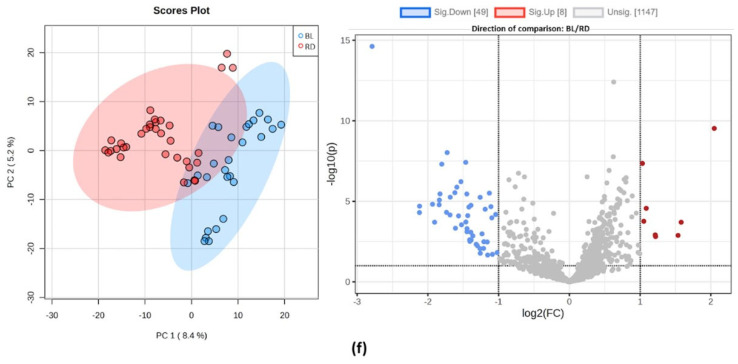
Principal component analysis score plots (left) and volcano plots (right) of groups analyzed in pairs: (**a**) yellow vs. blue, (**b**) yellow vs. green, (**c**) yellow vs. red, (**d**) blue vs. green, (**e**) green vs. red, (**f**) blue vs. red. In volcano plot graphs, the y-axis represents the *p*-value with a threshold of 0.1, and the x-axis represents the fold change (FC) with a threshold of 2.0.

After clustering, the groups were analyzed through univariate and multivariate methods in pairs. Therefore, all the samples within the same group were compared to each of the other groups, defined by color code as yellow (YW), blue (BL), green (GN), and red (RD); these groups consisted of 3, 9, 11, and 13 samples, respectively (Figure 2). This was applied to determine the features (*m/z*) statistically significant and differentiated among these groups.

As can be seen in Figure 2, most of the variance observed in the PCA score plots of the group pairs was attributed to PC1. The couples that presented a satisfactory separation were YW/BL, YW/GN, and BL/GN, which had explained variance values higher than 9% in PC1. On the other hand, it was observed that the group pairs that presented an explained variance lower than 8.5% in PC1 showed poor separation and overlapped. Therefore, it was concluded that PCA analysis could not differentiate the RD group from the BL and GN (Figure 2e,f). These results agree with those described in Figure 1, where no defined separation was observed in the first component (PC1) between the red, blue, and green groups.

From the pairwise PCA analyses, the following number of features (*m/z*) were up significantly: 131 for YW/BL, 95 for YW/GN, 79 for YW/RD, 59 for BL/GN, 34 for GN/RD, and 8 for BL/RD. In contrast, the following number of down-significant features (*m/z*) were found: 127 for YW/BL, 103 for YW/GN, 99 for YW/RD, 93 for BL/GN, 24 for GN/RD, and 49 for BL/RD. Regarding the number of features that differentiated the yellow, blue, and green groups, the BL/RD and GN/RD comparisons showed few significantly different features. Based on the overall results of PCAs and Volcano Plots, it was found that samples corresponding to the red group had chemical profiles like those of the green and blue groups; therefore, it was decided to exclude this group from the next part of the metabolic analysis.

Once the three groups of metabolic interest were selected, the increased and decreased features (*m/z*) were explored to find the corresponding metabolite responsible for the separation in the scoring plots. For this, fold change (FC) analysis, which describes how much a quantity changes from one group to another, was used [38]. To discriminate differential features (*m/z*), it was determined that fold change values greater than (or equal to) 2.0 are related to up-significant (+) features, while values less than (or equal to) 0.5 are related to down-significant (−) features. For example, for *m/z* 466.2002935, an FC value of 4.70 was determined for the GN/BL ratio indicating a higher amount of this analyte in the GN group compared to the BL group. For the GN/YW ratio, an FC value of 4.03 was found, which correlates with a higher amount of analyte in the GN group compared to the YW group. In contrast, in the YW/BL ratio, a value of 0.86 was found, which indicates that this analyte does not allow differentiation of the blue from yellow groups. Taking this into account, the ratio *m/z* 466.2002935 is recorded as increased for the green group (+) but decreased for the blue and yellow groups (−). Considering the FC results, only the following features (*m/z*) were found to be different: 16 up-significant metabolites and 15 down-significant metabolites for the yellow group, 9 up-significant metabolites and 10 down-significant metabolites for the blue group, and 6 up-significant metabolites and 3 down-significant metabolites for the green group (Table S2).

The features (*m/z*) with statistical significance were selected for presumptive identification through a comparison of their mass spectra and diagnostic fragment ions with those reported in the literature and databases. The assignment of the identity of each feature was made according to a list of possible candidates provided by the CEU mass mediator 3.0 search tool [39]. Table S2 shows the presumptive identification of the differential metabolites by comparing mass spectra, mass error of the molecular ion (less than or equal to 10 ppm), and the diagnostic fragment ions reported for each metabolite. Some features (*m/z*) were reported as unknown candidates since they did not match with any compound; however, due to their statistical significance, they were considered in the study.

In addition, Table S3 shows the identified metabolites as they were in higher or lower amounts for a particular group of samples. According to these metabolic analyses, quinolines, amino acids dipeptides (predominant), carbohydrates, phenols, thiols, methylpyridines, isoflavonoid, and phenylpropanoids were found to be present in the yellow group; cinnamic acid esters, monoterpenoids, diterpenoids, phenols and amino acids for the blue group; and carboxylic acids derivatives, flavonoids, and naphthalenes for the green group. In contrast, the following classes of compounds were found to be decreased: amino acids tripeptides, amino acids derivatives, alpha-amino acids, terpene lactones, naphthalenes, lipids, dicarboxylic acids, quinolines, and indoles for the yellow group; quinolines, isoquinolines, piperidines, nucleosides, benzenoids, monoterpenoids, and amino acids for the blue group; and diterpene, flavonoids, and alkaloids for the green group.

### 2.3. Relation of Physicochemical, Sensory, and Metabolic Analyses

In order to construct a global analysis and find possible representative characteristics that explain the clustering in the yellow, blue, green, and red groups at the PCA analysis, the physicochemical and sensory results were related to the metabolic results. Initially, it is important to note that the samples belonging to the yellow group corresponded to cocoa beans from the Cesar region, and the samples from the green group were collected from the Antioquia region. In contrast, the samples in the blue and red groups had a diverse origin of production: the blue group was constructed of seven samples from Antioquia, one from Santander, and one from Norte de Santander; and the red group was constructed of six samples from Antioquia, one from Santander, three from Cesar, and one from Magdalena. The wide variety, in terms of origin, of the cocoa samples in the last group may explain why it was not completely differentiated from the other groups leading to a similar chemical pattern to the blue and green groups.

**Figure 3:**
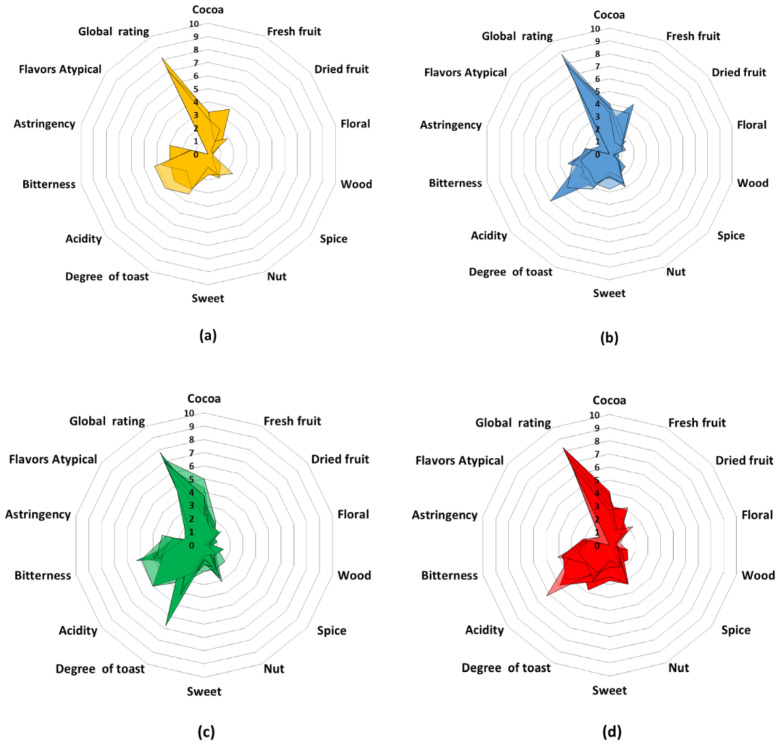
Radial plots of the sensory analysis of the cocoa samples grouped according to the results of the PCA: (**a**) yellow, (**b**) blue, (**c**) green, and (**d**) red groups.

Most of the chemical test results (except for cadmium concentration) are not different enough to differentiate this group of samples since all samples were in very similar overlapping ranges. The total phenolic content was in the following ranges: 103.4–151.5 (yellow), 88.9–122.8 (blue), 118.2–168.2 (green), and 88.1–143.4 (red), which were in accordance with metabolic results, where phenolic compounds were increased for the yellow and blue group. The ratio of theobromine/caffeine was in the following ranges: 1.9–2.1 (yellow), 1.7–4.8 (blue), 1.5–3.5 (green), and 1.4–3.8 (red). According to the metabolic results, alkaloids were decreased for the green group. Unlike these previous parameters, the cadmium concentration was able to differentiate the blue group from the others since five of the seven samples belonging to this group exceeded the permitted levels of this chemical element, while the samples of the other groups (except for one sample of the red group) were within the permitted values. It is interesting that these five samples came from Santander, Norte de Santander, and Urabá regions, where high content of Cd is characteristic of these regions. On the other hand, most of the sensory analyses had not characteristics that allowed differentiation of the groups, as can be seen in the radial diagrams (Figure 3).

The only differentiating characteristics were fresh fruit and atypical flavors. The fresh fruit values were in the following ranges: 1.1–3.8 (yellow), 0.3–4.4 (blue), 0.0–2.0 (green), and 0.6–3.2 (red). Here, the green group differs from the others because it had lesser notes of citrus fruits, tropical fruits, and berries. According to the metabolic results, diterpenes and flavonoids (associated with this type of fruit) were decreased for the green group and increased in the yellow and blue groups. As for atypical flavors, the samples of the yellow group were the only ones that did not present any strange flavor, and one sample of the green group was the only one with the highest value in this characteristic. This could probably be related to the high content of naphthalene compounds for the green group according to the presumptive metabolic identification.

**Figure 4:**
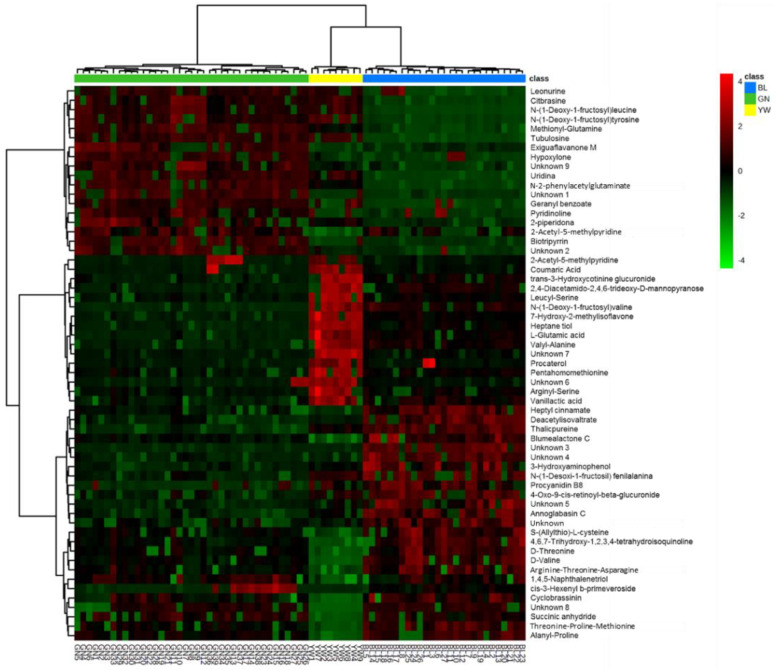
Heat map of the putatively identified metabolites for each group of samples. On the right side, the labels of the metabolites identified are shown, and at the bottom, the names of the samples are listed.

During the cocoa fermentation process, different metabolic changes take place, and they will determine the cocoa quality. This process begins with the growth of microorganisms, which convert sugars in the pulp surrounding the beans into ethanol. This ethanol then begins to oxidize into acetic acid (aerobic conditions), lactic acid (anaerobic conditions), carbon dioxide, and water, causing an increase in temperature and decomposition of the pulp. This increase in temperature and acetic acid concentration kills the cocoa bean. Death causes the breakdown of the cell walls allowing complex chemical changes to occur thanks to the mixture of substrates and enzymes that cause the flavor and color of the chocolate to develop [40]. Among the most important changes are oxidation of polyphenols (epicatechin, catechin, procyanidin, cyanidin, leucocyanidin), formation of tannins (associated with astringent flavors), protein catabolism (responsible for the release of hydrophobic amino acids and hydrophilic oligopeptides associated with the generation of aromatic notes), fermentation, and degradation of sugars and organic acids, Maillard reactions of amino acids and sugars (during roasting stage) producing aldehydes, esters, alcohols, ketones, and pyrazines, related to nutty, fruity and floral notes [10]. To visualize the behavior of the differential metabolites for each group, a heat map was constructed relating the intensities of each putative compound identified (Figure 4). Metabolites with significantly higher abundance are represented in red, while metabolites with lower abundance are green-colored. 

In the heat map plot (Figure 4), the samples of the yellow group were characterized by the increased presence of dipeptides (consisting of leucine, serine, valine, and phenyl alanine), quinolines, carbohydrates, phenols, thiols, and isoflavonoids, while amino acid derivatives (containing valine, alanine, and proline), terpenes (associated with flowers), lipids, naphthalene compounds, dicarboxylic acids, quinolines, indoles, and tripeptides (consisting of threonine, proline, methionine, arginine and asparagine), which were probably converted into dipeptides during enzymatic digestion, were decreased. The samples of this group had a wide diversity of differential compounds that are of great nutritional interest, and the peptide derivatives can be used to study their properties to be useful for different industries (pharmaceuticals, cosmetics, among others). The blue group samples were rich in cinnamic acid esters (product of Maillard reactions), monoterpenoids (associated with citrus fruits), phenols, methionine, and glutamine peptides. These samples had a decreased abundance of quinolines, benzenoid piperidines, amino acid monoterpenes of leucine, threonine, and glutamine. However, it is interesting that the samples from the blue group were the only ones with a decrease in uridine nucleoside, which plays a role in the galactose glycolysis pathway [41]. Finally, the samples of the green group were the ones with the least variety of compounds, both increased and decreased. They were rich in carboxylic acid derivatives, flavonoids, and naphthalene compounds and poor in diterpenes and alkaloids. It is recommended to perform a targeted metabolomics approach using flavor and aroma reference standards as a complement to the sensory analyses to validate the results presented here.

## 3. Materials and Methods

### 3.1. Sampling and Sensorial Analysis

A total of 36 samples of cocoa beans used in this study were provided from farms coming from the regions of Antioquia, Cesar, Magdalena, Norte de Santander, and Santander in Colombia; the grains were fermented for 6 days in wooden crates of 60 cm × 60 cm × 60 cm covered with banana leaves. Sampling was performed by trained technicians provided by the CAESCA work team in the macro-project of Colombia + Competitiva PC+C042-017 program of the Swiss Embassy in Colombia, which is part of the Swiss Program for Economic Development Cooperation in Colombia (SECO). The sensory tests were performed by a set of professional testers, who followed the ISO 13299:2016 standard methodology to the guidelines for establishing a sensory profile in cocoa beans, the different criteria of aroma, basic tastes, trigeminal sensation, and atypical flavors were evaluated, and the intensity of this attributes was scored for each descriptor, using a scale of categories from 0 “imperceptible” to 10 “intense” [31].

### 3.2. Quantification of Cadmium Content 

The cadmium content determination was carried out at the chemical industrial consultation laboratory of Universidad Industrial de Santander using a methodology according to the standard protocol AOAC 999.10 of 2005 with slight modifications [42]. This interpretation was accredited under scope 21-LAB-031 of the Colombian National Accreditation Agency. Briefly, the cocoa beans were dried for 4 h at 70 °C, ground, and sieved. About 0.25 g of powder was weighed, and microwave-assisted digestion was performed in a 30% (*v*/*v*) hydrogen peroxide, hydrochloric acid, and nitric acid solution. Finally, the extract was filtered, and the total cadmium content was determined by flame or graphite furnace atomic absorption spectroscopy. 

### 3.3. Metabolite Extraction and Sample Preparation

At first, each cocoa bean was subjected to a defatting process, as described by Mayorga et al. [13]. For this, the samples were ground, and 1.5 g of powder were suspended in 12 mL of *n*-hexane; the mixture was then stirred for 20 min at room temperature and centrifuged at 10,000× *g* for 10 min. The supernatant was then discarded, and the solid was preserved and dried in an oven at 40 °C for 48 h.

The extraction of non-volatile metabolites was performed by solid-phase extraction methodology as described by Menguy et al., 2009 [43]. Briefly, 100 mg of defatted cocoa powder was dissolved in 1.5 mL of ice-cold acetonitrile 50% (*v/v*). The solution was placed in an ultrasonic bath (Elma, Singen, Germany) at 30 °C for 30 min, then centrifuged at 10,000× *g* for 10 min, 1 mL of the organic extract was dried in a Savant Speed Vac SPD120 vacuum concentrator (Thermo Fisher Scientific, Asheville, NC, USA). The dried extract was resuspended in 1 mL of ultra-pure water, sonicated, and passed twice through an SPE-C18 cartridge. The content of the cartridge was eluted with 1 mL (×11) of 50% aqueous methanol (*v/v*), and the metabolic extract obtained was used for polyphenol quantification and metabolic profiling.

### 3.4. Total Polyphenols Content

Polyphenols were determined using the Folin–Ciocalteu colorimetric method. It was carried out following the process described by Al-Owaisi et al., 2014 [44] Briefly, 860 μL of 7.5% (*m/v*) sodium carbonate solution was mixed with 430 μL of Folin–Ciocalteau 2.0 M reagent diluted 1:10 with deionized water, and 150 μL of the sample described in Section 3.3; the mix was shaken vigorously. The mixture was incubated for 1 h at room temperature and protected from light, and its absorbance was recorded at a wavelength of 765 nm in a UV-1800 spectrophotometer (Shimadzu, Tokyo, Japan). To construct the calibration curve, serial dilutions of gallic acid (purity > 98%, Sigma-Aldrich, Saint Louis, MO, USA) standard at a concentration of 30, 45, 60, 60, 85, 100, and 120 ppm were prepared. Finally, the results were expressed in terms of mg of gallic acid per gram of dried cocoa bean sample (mg G.A/g sample).

### 3.5. Chromatographic Analysis of Metabolic Extracts

Metabolic profiling was performed following the methodology described by Ortega et al. [45]. Briefly, the methanolic extracts were injected into a UPLC-MS/MS system (DionexTM UltimateTM 3000 (Thermo Scientific, Sunnyvale, CA, USA) Orbitrap (Exactive Plus, Thermo Scientific, Sunnyvale, CA, USA)) equipped with an ESI interface operated at 3500 V in the positive mode of ionization, and the nebulization gas was nitrogen at a temperature of 375 °C. Chromatographic separation was performed on an Acclaim RSLC 120 C18 column (2.1 mm × 150 mm × 2.2 µm). Mobile phases were A: 2% acetic acid in water and B acetonitrile. The elution gradient was settled as follows: 0–2 min, 0% B; 2–18 min, 0–100% B; and 18–20 min, 100% B. The procedure described by Ortega et al. was used. Briefly, an aliquot of 500 μL of the metabolic extract was analyzed in the UHPLC-ESI-Orbitrap-HRMS instrument. A DionexTM UltimateTM 3000 UHPLC (Thermo Scientific, Sunnyvale, CA, USA), equipped with a degasser (SRD-3400), a binary gradient pump (HPG3400RS), an autosampler (WPS 300TRS), and a thermostated unit for the column (TCC 3000). Chromatographic separation was performed on an Acclaim RSLC 120 C18 column (2.1 mm × 150 mm × 2.2 µm). Mobile phases were A: 2% acetic acid in water and B acetonitrile. The elution gradient was settled as follows: 0–2 min, 0% B; 2–18 min, 0–100% B; and 18–20 min, 100% B. The sample volume injected was 2.5 μL, and the flow rate was 300 μL/min. UHPLC was coupled to a high-resolution mass spectrometer with an Orbitrap-type ion current detection system (Exactive Plus, Thermo Scientific, Sunnyvale, CA, USA) through an electrospray interface (HESI-II), operated in positive ion mode at 350 °C, a capillary voltage of + 4000 V and a temperature of 320 °C. The orbitrap mass analyzer was operated in full scan mode (Full MS Scan) with a resolution of 30,000 m/m in the positive mode of ionization. The molecular ions were sent for fragmentation to the HCD (higher-energy collisional dissociation cell) at the energies of 10, 20, 30, and 40 eV. The dynamic range of masses was from 80 to 1000 *m/z*. For the instrumental analysis based on UHPL-HRMS, each of the 36 samples studied had a technical triplicate. From the preliminary treatment of these data and the statistical analysis described in the following section, the metabolic profiles of the samples were obtained. 

The determination of the amount of caffeine and theobromine and T/C ratio of cocoa beans was found from the quantification by % relative amount of the metabolic profiles of the samples. For this, the peaks of both methyl-xanthines were identified in the full ion chromatogram, and the intensity of their peaks was extracted.

### 3.6. Data Analysis

Once the metabolic profiles were acquired, the files were transformed into MzXML by using the tool MS Convert 3.0 from Proteowizzard. Chromatogram alignment, background noise subtraction, and feature detection were carried out at the online platform XCMS (Version 3.7.1) https://xcmsonline.scripps.edu/ (accessed on 1 April 2022). The data were filtered according to their coefficient of variation (equal to or less than 30%), and data matrices were generated. Principal component analysis (PCA) was applied to all the data to determine the clustering in the samples. Normalization was performed by the total sum of intensities from the mass spectra, logarithmic transformation, and auto-scaling of data were carried out with MetaboAnalyst software (Version 5.0.) (https://www.metaboanalyst.ca/MetaboAnalyst/home.xhtml) (accessed on 15 July 2022).

Differences in the metabolic profiles of the sample groups established by multivariate analysis were further explored by univariate analysis [46]. For this purpose, all samples within a single group were treated as if they were replicates of a single sample. Thus, fold change analysis (FC) and *t*-test were applied in pairs of sample groups to determine *m/z* discriminant features. A metabolite was considered statistically significant if its *p*-value was equal to or less than 0.05 and its FC was higher than 2 or lower than 0.5. Putative identification of the statistically significant features was performed by a search for possible matches using the CEU Mass-Mediator platform (version 3.0) (http://ceumass.eps.uspceu.es/) (accessed on 19 September 2022) [39]. Then, flavor-associated metabolites were presumptively identified by comparing the characteristic ions of their mass spectra with those reported in open-access mass spectrometry libraries such as Kegg, HMDB, LipidMaps, Metlin, NP Atlas, KNApSAcK, mass bank, and MINE. 

## 4. Conclusions

The present study revealed that a non-targeted metabolomics approach based on UHPLC/HRMS combined with multivariate and univariate statistical analysis was successfully used to discriminate cocoa bean samples and identify differential chemical markers related to physicochemical and sensory properties associated with cocoa quality. Furthermore, although the chemical (cadmium and polyphenols content) and sensory characterization of the 36 samples were quite similar, the physical analyses, together with the metabolomics approach, allowed us to find differences and classify the samples into four groups or clusters. In the metabolic composition of the samples, alkaloids, flavonoids, terpenoids, peptides, quinolines, and sulfur compounds were identified as discriminants between sample groups. It is recommended to perform a targeted metabolomics approach using flavor and aroma reference standards as a complement to the sensory analyses and to the present study. This investigation represents an alternative method for the quality control of cocoa beans, which could complement the regulatory analyses performed by tasters and allow a better distinction between fine and ordinary cocoa.

## Figures and Tables

**Table 1:** Results of the physical characterization of the cocoa samples.

I.D	Sample Identifier	Grain Index (g/Grain)	Total Defective Grains	Fermentation Degree	Cracking Degree	Global Rating
01	MAN 1	1.04	7.5%	97.0%	69.5%	8.1
02	MAN 2	0.88	11.1%	99.0%	80.5%	7.9
03	MAN 3	0.90	11.0%	94.0%	76.0%	8.1
04	POR 1	1.28	4.0%	96.5%	86.5%	8.9
05	REG	1.50	6.0%	96.0%	86.5%	9.2
06	SAR	1.38	12.0%	99.0%	89.0%	9.1
07	MAK	1.13	13.0%	97.0%	86.5%	8.7
08	URA 1	1.44	10.0%	96.0%	87.0%	9.1
09	URA 2	1.50	16.0%	96.5%	91.5%	9.1
10	POR 2	1.53	10.0%	97.5%	93.0%	9.3
11	SDR	1.17	36.5%	84.5%	89.5%	8.0
12	URA 3	1.21	24.5%	99.5%	87.5%	8.6
13	CCN 1	1.43	3.0%	65.5%	96.0%	8.7
14	L8T7	1.38	4.5%	61.0%	92.5%	8.5
15	CCN 2	1.68	2.5%	70.0%	98.0%	8.9
16	CCN 3	1.50	14.0%	78.5%	92.0%	8.7
17	CCN 4	1.50	13.0%	66.5%	93.5%	8.6
18	CCN 5	1.50	12.0%	80.5%	94.0%	8.9
19	CCN 6	1.20	9.0%	94.0%	80.0%	8.6
20	CCN 7	1.26	8.0%	94.6%	87.5%	8.7
21	CCN 8	1.25	7.5%	90.5%	93.5%	8.8
22	CCN 9	1.53	10.0%	90.0%	90.5%	9.0
23	CCN 10	1.62	4.0%	86.5%	88.0%	9.2
24	CCN 11	1.42	11.5%	77.5%	93.0%	8.6
25	CCN 12	1.24	11.0%	94.5%	79.0%	8.6
26	CCN NO	1.43	0.0%	83.5%	89.0%	9.0
27	MAN 4	1.09	13.0%	93.5%	89.5%	8.6
28	MAN 5	1.14	11.0%	88.5%	87.0%	8.5
29	SV 1	1.42	4.5%	57.5%	88.5%	8.3
30	SV 2	1.64	5.0%	94.0%	95.5%	9.3
31	CCN 13	1.48	17.5%	75.0%	94.5%	8.6
32	MEZ 1	1.85	10.0%	67.0%	79.0%	8.7
33	MAN 6	1.38	5.0%	92.5%	81.0%	8.7
34	MON	1.02	10.0%	98.0%	89.0%	8.7
35	ICS	1.22	6.5%	76.5%	85.5%	8.4
36	SNM	1.68	9.5%	95.0%	87.0%	9.3

**Table 2:** Results of cadmium concentration, total phenolic content, and theobromine/caffeine ratio.

I.D	Sample Identifier	Total Phenolic Content (mg G.A/ g Sample)	Total Cadmium Content (mg/kg in Almonds)	Relative Amount (%) of Caffeine (C)	Relative Amount (%) of Theobromine (T)	T/C
01	MAN 1	126.8	0.4208	10.39	20.47	1.97
02	MAN 2	103.4	0.2406	9.07	19.36	2.13
03	MAN 3	151.5	0.5403	11.19	19.76	1.77
04	POR 1	90.1	0.5760	10.87	13.94	1.28
05	REG	96.8	0.4380	7.95	15.16	1.91
06	SAR	112.8	8.576	4.35	11.97	2.75
07	MAK	100.0	0.4130	9.94	11.14	2.75
08	URA 1	88.9	1.694	7.62	11.82	1.55
09	URA 2	115.4	1.491	7.41	13.16	1.78
10	POR 2	100.7	0.3600	11.84	13.59	1.15
11	SDR	119.8	7.069	7.60	11.47	1.51
12	URA 3	122.8	1.379	5.66	10.42	1.84
13	CCN 1	128.0	0.3611	7.33	18.83	2.57
14	L8T7	153.1	0.6477	6.22	16.67	2.68
15	CCN 2	131.0	0.5370	10.20	16.75	1.64
16	CCN 3	141.4	0.5352	8.67	17.95	2.07
17	CCN 4	136.5	0.3593	6.94	16.80	2.42
18	CCN 5	118.2	0.2927	6.55	15.26	2.33
19	CCN 6	140.6	0.4177	5.43	13.30	2.45
20	CCN 7	141.3	0.1806	4.69	14.59	3.11
21	CCN 8	124.1	0.2934	6.36	15.35	2.41
22	CCN 9	138.5	0.4642	6.45	15.64	2.43
23	CCN 10	168.2	0.5400	6.76	17.69	2.62
24	CCN 11	146.9	0.6878	7.42	18.08	2.44
25	CCN 12	147.1	0.4817	4.80	14.63	3.05
26	CCN NO	121.4	0.2903	10.97	17.22	1.57
27	MAN 4	120.8	2.934	10.00	17.57	1.76
28	MAN 5	134.5	1.582	9.02	14.82	1.64
29	SV 1	118.9	0.9624	8.91	15.37	1.72
30	SV 2	88.1	0.7161	9.93	14.83	1.49
31	CCN 13	143.4	0.4819	8.45	12.86	1.52
32	MEZ 1	117.3	0.6325	8.48	16.41	1.93
33	MAN 6	122.5	0.1190	10.29	18.73	1.82
34	MON	91.7	10.130	3.30	11.97	3.62
35	ICS	143.4	0.3570	7.17	17.40	2.43
36	SNM	124.7	0.3540	6.58	16.14	2.45

**Table 3:** Results of aroma descriptors to sensory test evaluated for cocoa samples.

I.D	Sample Identifier	Cocoa	Fresh Fruit	Dried Fruit	Floral	Wood	Spice	Nut	Sweet	Degree of Toast
01	MAN 1	3.2	3.8	1.3	0.5	0.2	1.2	2.0	1.5	3.0
02	MAN 2	3.3	1.1	1.9	0.3	0.4	0.4	1.7	1.6	2.9
03	MAN 3	2.7	2.1	0.3	0.4	0.4	2.4	1.8	1.0	3.4
04	POR 1	2.6	1.9	1.3	1.3	0.1	0.9	2.1	1.1	2.9
05	REG	3.0	0.3	1.7	0.4	0.6	0.1	2.4	1.1	3.0
06	SAR	2.6	4.4	1.4	0.6	0.5	1.6	1.8	1.4	2.4
07	MAK	3.6	1.7	1.4	0.4	0.1	0.0	2.7	1.9	2.3
08	URA 1	3.8	1.5	1.3	0.5	0.3	1.0	2.5	2.8	2.6
09	URA 2	4.0	2.2	1.5	0.7	0.8	0.7	2.7	1.7	2.5
10	POR 2	3.7	1.7	1.4	0.9	0.4	0.7	2.9	1.7	2.9
11	SDR	3.6	3.6	1.3	0.4	0.3	0.9	2.1	1.4	3.1
12	URA 3	3.3	1.0	1.1	1.1	0.3	0.9	2.6	1.8	2.6
13	CCN 1	4.3	1.3	1.3	0.3	1.1	1.1	3.1	1.3	3.0
14	L8T7	2.9	0.0	0.1	0.0	0.1	0.4	1.3	0.8	5.8
15	CCN 2	3.3	1.8	0.8	0.4	0.0	0.6	2.8	1.4	3.5
16	CCN 3	3.6	0.9	0.9	1.0	0.5	0.9	2.6	1.4	4.0
17	CCN 4	5.0	2.0	1.0	0.0	1.0	2.0	2.0	2.0	3.0
18	CCN 5	3.1	1.8	0.9	0.4	0.4	0.9	1.9	1.5	3.1
19	CCN 6	2.8	1.5	1.5	0.5	0.5	1.0	1.8	1.5	3.0
20	CCN 7	2.7	1.1	0.9	0.1	0.0	1.0	2.0	1.1	2.7
21	CCN 8	2.6	0.8	1.1	0.4	1.5	1.1	1.5	1.1	6.8
22	CCN 9	3.7	0.9	1.0	0.1	0.4	0.7	1.9	1.1	3.4
23	CCN 10	3.7	0.2	1.0	0.0	0.3	0.3	2.2	0.7	4.2
24	CCN 11	2.7	0.5	1.7	0.3	0.5	0.2	2.2	1.0	3.7
25	CCN 12	2.3	1.2	1.2	0.0	0.3	1.2	0.8	0.8	2.5
26	CCN NO	4.1	1.0	1.8	0.4	0.6	1.1	3.0	1.8	2.9
27	MAN 4	2.5	2.5	1.3	0.5	0.5	1.3	2.2	1.5	3.0
28	MAN 5	3.3	1.7	1.4	0.1	1.4	1.8	1.6	1.4	2.7
29	SV 1	3.7	1.8	0.8	0.4	0.8	1.3	3.3	2.7	3.8
30	SV 2	3.1	1.7	1.7	0.4	0.1	0.9	3.0	1.4	3.0
31	CCN 13	3.4	0.6	1.7	0.8	1.3	0.7	3.2	2.4	3.1
32	MEZ 1	3.0	3.0	0.9	0.4	0.0	0.4	1.7	1.1	3.1
33	MAN 6	2.5	2.3	1.7	0.2	0.7	0.8	1.5	1.2	2.5
34	MON	2.7	3.2	1.0	0.5	0.5	1.0	1.8	1.2	2.2
35	ICS	2.6	1.1	1.3	0.6	0.6	0.4	1.9	1.1	2.9
36	SNM	3.8	1.3	2.3	0.5	0.5	1.0	1.8	1.7	2.3

**Table 4:** Results of descriptors in basic tastes, trigeminal sensation, atypical flavors, and overall sensory score of the cocoa samples.

I.D	Identification of the Sample	Acidity	Bitterness	Astringency	Atypical Flavors	Global Rating
01	MAN 1	3.3	3.0	3.0	0.0	8.2
02	MAN 2	2.1	3.7	1.4	0.0	7.0
03	MAN 3	4.2	4.2	1.3	0.0	7.3
04	POR 1	3.3	2.5	2.0	0.0	7.4
05	REG	2.0	3.4	1.6	1.0	6.3
06	SAR	6.0	2.3	1.6	0.0	8.4
07	MAK	2.1	2.4	1.3	0.0	7.3
08	URA 1	2.9	1.8	1.9	0.0	8.8
09	URA 2	2.2	3.0	1.8	0.0	7.7
10	POR 2	2.6	2.3	1.4	0.0	8.0
11	SDR	4.3	3.0	1.4	0.0	8.3
12	URA 3	2.1	2.4	1.4	0.0	7.9
13	CCN 1	2.4	4.7	2.3	0.0	6.5
14	L8T7	1.5	5.3	1.5	1.9	4.9
15	CCN 2	3.4	4.0	2.4	0.0	7.1
16	CCN 3	1.9	3.8	1.9	0.0	7.0
17	CCN 4	5.0	4.9	3.0	0.0	7.2
18	CCN 5	2.5	3.1	2.3	0.5	7.4
19	CCN 6	3.8	3.0	1.6	0.0	7.8
20	CCN 7	2.6	3.3	2.4	0.0	7.1
21	CCN 8	2.4	3.4	2.2	0.0	6.0
22	CCN 9	2.3	4.0	2.1	0.0	7.1
23	CCN 10	1.5	3.7	3.3	0.0	7.0
24	CCN 11	1.8	3.3	2.5	0.0	7.0
25	CCN 12	5.0	3.2	1.7	1.3	5.3
26	CCN NO	2.1	3.4	1.4	0.0	8.3
27	MAN 4	3.8	3.0	2.0	0.0	7.7
28	MAN 5	3.7	3.3	1.7	0.0	6.3
29	SV 1	2.7	3.8	2.1	0.0	6.8
30	SV 2	3.6	3.0	2.0	0.0	7.7
31	CCN 13	2.3	4.1	1.8	0.0	7.8
32	MEZ 1	4.9	3.0	1.6	0.0	7.9
33	MAN 6	4.3	3.7	1.7	0.0	7.5
34	MON	6.2	3.0	1.7	1.0	6.7
35	ICS	2.3	3.8	2.1	1.0	6.5
36	SNM	2.2	2.5	1.7	0.0	8.3

## Data Availability

Not applicable.

## Supplementary Materials

The following supporting information can be downloaded at: https://www.mdpi.com/article/10.3390/molecules28114467/s1, Table S1: Physicochemical characterization of cocoa samples; Table S2: Presumptive identification of differential metabolites based on mass spectrum analysis and retention time. Table S3: Identified metabolites to be up-modulated (+) and down-modulated (−) for each set of samples

## Sample Availability

Samples of the compounds are not available from the authors.
